# Pepper aldehyde dehydrogenase CaALDH1 interacts with *Xanthomonas* effector AvrBsT and promotes effector-triggered cell death and defence responses

**DOI:** 10.1093/jxb/erv147

**Published:** 2015-04-06

**Authors:** Nak Hyun Kim, Byung Kook Hwang

**Affiliations:** Laboratory of Molecular Plant Pathology, College of Life Sciences and Biotechnology, Korea University, Seoul 136-713, Republic of Korea

**Keywords:** Aldehyde dehydrogenase, cell death, effector AvrBsT, pepper, plant defence, *Xanthomonas campestris* pv. *vesicatoria*.

## Abstract

*Xanthomonas* type III effector AvrBsT induces hypersensitive cell death and defence responses in pepper (*Capsicum annuum*) and *Nicotiana benthamiana*. Little is known about the host factors that interact with AvrBsT. Here, we identified pepper aldehyde dehydrogenase 1 (CaALDH1) as an AvrBsT-interacting protein. Bimolecular fluorescence complementation and co-immunoprecipitation assays confirmed the interaction between CaALDH1 and AvrBsT *in planta*. CaALDH1:smGFP fluorescence was detected in the cytoplasm. *CaALDH1* expression in pepper was rapidly and strongly induced by avirulent *Xanthomonas campestris* pv. *vesicatoria* (*Xcv*) Ds1 (*avrBsT*) infection. Transient co-expression of *CaALDH1* with *avrBsT* significantly enhanced *avrBsT*-triggered cell death in *N. benthamiana* leaves. Aldehyde dehydrogenase activity was higher in leaves transiently expressing *CaALDH1*, suggesting that CaALDH1 acts as a cell death enhancer, independently of AvrBsT. *CaALDH1* silencing disrupted phenolic compound accumulation, H_2_O_2_ production, defence response gene expression, and cell death during avirulent *Xcv* Ds1 (*avrBsT*) infection. Transgenic *Arabidopsis thaliana* overexpressing *CaALDH1* exhibited enhanced defence response to *Pseudomonas syringae* pv. *tomato* and *Hyaloperonospora arabidopsidis* infection. These results indicate that cytoplasmic CaALDH1 interacts with AvrBsT and promotes plant cell death and defence responses.

## Highlight

Pepper aldehyde dehydrogenase CaALDH1 interacts with *Xanthomonas* type III effector AvrBsT and plays a significant role for positive regulation of effector-triggered cell death and defence responses in plants.

## Introduction

Microbial pathogens attack plants to acquire nutrients for growth, development, and reproduction (Dangl *et al.*, 2013). Pathogens that breach the protective waxy cuticular leaf surface encounter an immune system that specifically recognizes selected pathogens and altered-self molecules generated during pathogen invasion (Eulgem, 2005; Choi *et al.*, 2012; Dangl *et al.*, 2013). The first tier of the plant immune system contains pattern-recognition receptors (PRRs) localized on the cell surface that bind to evolutionarily conserved pathogen-associated molecular patterns (PAMPs) (Jones and Dangl, 2006; Dodds and Rathjen, 2010; Monaghan and Zipfel, 2012). PRRs bind to the pathogen-derived ligand, become activated, and trigger an intracellular signalling cascade that drives transcriptional reprogramming and defence molecule biosynthesis to inhibit pathogen colonization (Monaghan and Zipfel, 2012). Plant bacterial pathogens utilize the type III secretion system to deliver effector proteins that subvert PRR-triggered defence responses (Kim *et al.*, 2010; Block and Alfano, 2011). The second tier of the plant immune system contains intracellular nucleotide-binding leucine-rich repeat (NLR) receptors (Maekawa *et al.*, 2011). NLR proteins recognize specific pathogen effectors either via direct interaction with the effector protein (Dodds *et al.*, 2006) or via recognition of pathogen effector-mediated modifications (van der Hoorn and Kamoun, 2008).

The hypersensitive response (HR)-like cell death that occurs during pathogen attack is an integral part of plant immune systems, and is one of the most dramatic displays of programmed cell death (PCD) in plants (Bozhkov and Lam, 2011). The hallmarks of HR include rapid oxidative burst, production of reactive oxygen species (ROS) such as H_2_O_2_, and ion fluxes across the plasma membrane before cell death (Lamb and Dixon, 1997; Morel and Dangl, 1997). ROS is intimately related to plant cell death and defence signalling (Torres *et al.*, 2006; Van Breusegem and Dat, 2006). ROS directly inhibits pathogen growth, stimulates cell wall cross-linking, and triggers defence- and stress-response gene expression (Lamb and Dixon, 1997; Skopelitis *et al.*, 2006).

*Xanthomonas campestris* pv. *vesicatoria* (*Xcv*) strain Bv5-4a secretes type III effector protein AvrBsT, which induces hypersensitive cell death and strong defence responses in pepper (*Capsicum annuum*) and *Nicotiana benthamiana* (Orth *et al.*, 2000; Escolar *et al.*, 2001; Kim *et al.*, 2010). AvrBsT is a member of the YopJ/AvrRxv family in *Xcv* (Lewis *et al.*, 2011). AvrBsT possesses acetyltransferase activity and acetylates ACIP1 (for *ACETYLATED INTERACTING PROTEIN1*), an unknown protein from *Arabidopsis* (Cheong *et al.*, 2014). ACIP1 is proposed to function in the defence machinery required for anti-bacterial immunity. However, the molecular mechanisms and host factors involved in AvrBsT-triggered cell death are not completely elucidated.

Aldehyde dehydrogenases (ALDHs; EC 1.2.1.3) catalyse the conversion of aldehydes to the corresponding carboxylic acids and reduce NAD+ or NADP+. Human ALDHs are extensively characterized and categorized as cytosolic ALDH1 and mitochondrial ALDH2 that are involved primarily in ethanol metabolism (Hsu *et al.*, 1988; Hsu *et al.*, 1989). More than 550 *ALDH* genes have been identified in mammals, insects, bacteria, yeast, and plants (Sophos and Vasiliou, 2003). Some ALDHs oxidize specific substrates, whereas others accept a broad range of substrates (Yoshida *et al.*, 1998). Comprehensive genetic information has led to the establishment of the ALDH Gene Nomenclature Committee that defines specific characteristic criteria for ALDH proteins (Vasiliou *et al.*, 1999). Family 1 ALDHs include the original Class 1 ALDHs that are targeted to the cytosol. Family 2 ALDHs include the mitochondrial Class 2 ALDHs. Family 1 and 2 ALDHs play a major role in detoxification of ethanol-derived acetaldehyde (Wang *et al.*, 1998). Aldehydes are produced from lipid peroxidation and are toxic because of their chemical reactivity. Family 3 ALDHs detoxify aldehydes formed during lipid peroxidation (Lindahl and Petersen, 1991).

ALDHs are involved in plant growth, development, and stress responses (Sunkar *et al.*, 2003; Kotchoni *et al.*, 2006, 2010; Shin *et al.*, 2009). Maize (*Zea mays*) mitochondrial ALDH2B2 (also known as *rf2a*) is essential for normal anther development and male fertility (Liu *et al.*, 2001; Liu and Schnable, 2002). Rice (*Oryza sativa*) OsALDH7 function is crucial for seed maturation and viability (Shin *et al.*, 2009). A well-characterized stress-responsive ALDH is the osmotic stress-inducible betaine aldehyde dehydrogenase, which catalyses synthesis of the osmolyte glycine betaine using betaine aldehyde as the substrate (Chen and Murata, 2002). *ALDH3I1* and *ALDH7B4* overexpression in *Arabidopsis* significantly enhances tolerance to drought, salinity, and oxidative stress (Kotchoni *et al.*, 2006). The steady-state *ALDH21A1* transcript level in *Tortula ruralis* is elevated in response to dehydration, NaCl, abscisic acid (ABA), and UV irradiation (Chen *et al.*, 2002). Two *Arabidopsis ALDH* genes are upregulated by dehydration, high salinity, and cold (Seki *et al.*, 2002). Two barley *ALDHs* are upregulated by drought stress (Ozturk *et al.*, 2002). ALDH protein accumulates to high levels in the rice lesion mimic mutant *cdr2,* suggesting a role for ALDH in plant programmed cell death (PCD) and defence signalling (Tsunezuka *et al.*, 2005).

In this study, we isolated and identified pepper aldehyde dehydrogenase CaALDH1 as an AvrBsT-interacting protein using yeast two-hybrid screening. CaALDH1:smGFP (soluble-modified green fluorescent protein) fluorescence was detected in the cytoplasm. Heterologous transient co-expression of *CaALDH1* and *avrBsT* in *N. benthamiana* leaves significantly enhanced *avrBsT*-triggered cell death. Cell death promotion by *CaALDH1* expression depended on CaALDH1 aldehyde dehydrogenase activity. *CaALDH1* expression in pepper was rapidly and strongly induced by avirulent *Xcv* Ds1 (*avrBsT*) infection. *CaALDH1* silencing in pepper disrupted *Xcv*-induced aldehyde dehydrogenase activity, H2O2 accumulation, and cell death response, and reduced resistance to avirulent *Xcv* infection. Defence response gene expression also was reduced by *CaALDH1* silencing. *CaALDH1* overexpression (OX) in transgenic *Arabidopsis* plants reduced susceptibility to *Pseudomonas syringae* pv. *tomato* and *Hyaloperonospora arabidopsidis* Noco2 infection. These results demonstrate the functional importance of pepper aldehyde dehydrogenase CaALDH1 for regulation of AvrBsT-triggered cell death and defence responses in plants.

## Materials and methods

### Plant materials and growth conditions

Pepper (*Capsicum annuum* L. cv. Nockwang) and *Nicotiana benthamiana* were planted in plastic pots containing a soil mix (loam:perlite:vermiculite, 3:1:1, v/v/v) at 28°C with a long-day photoperiod (16h light/8h dark) with a light intensity of 100 μmol photons m^-2^ s^-1^. *Arabidopsis thaliana* ecotype Col-0 were grown in pots containing vermiculite, peat moss, and perlite (1:1:0.5, v/v/v) at 24°C, 60% relative humidity and 130 μmol photons m^-2^ s^-1^ with a 16h light photoperiod in a growth chamber.

### Yeast two-hybrid assay

The *avrBsT* open reading frame (ORF) was cloned into *Bam*HI/*Hind*III sites of the pGBKT7 vector. The yeast prey library was generated from a pepper cDNA library by ligating cDNA inserts into the pGADT7 vector. The constructs were co-transformed into yeast strain AH109 and plated onto synthetic dropout (SD) –histidine, ↕leucine, ↕tryptophan (↕HLT) media (Ito *et al.*, 1983). Approximately 50 000 colonies from the SD-HLT media were transferred onto selection media (SD)↕adenine, ↕histidine, ↕leucine, ↕tryptophan (↕AHLT). Plasmids were extracted from the surviving yeast colonies and used to transform *E. coli.* Colonies carrying pGADT7 were selected on Luria-Bertani (LB) media containing 100mg l^-1^ ampicillin. Isolated plasmids were sequenced, and sequence homology was analysed using Genbank BLAST tools (http://blast.ncbi.nlm.nih.gov/Blast.cgi).

### Bimolecular fluorescence complementation assay

Bimolecular fluorescence complementation (BiFC) analyses were conducted as described previously (Waadt *et al.*, 2008). To generate the BiFC constructs, the *CaALDH1* coding region without termination codons was PCR-amplified and subcloned into the binary vectors pSPYNE (*Xba*I/*Xho*I) and pSCYCE (*Xba*I/*Xho*I) under control of the cauliflower mosaic virus 35S promoter (see Supplementary Table S1 for the oligonucleotide sequences). pSPYNE:*avrBsT* and pSPYCE:*avrBsT* were generated as described previously (Kim *et al.*, 2014). *AvrBsT* and *CaALDH1* fusion constructs were co-expressed in *N. benthamiana* leaves by infiltrating *A. tumefaciens* strain GV3101 carrying each construct (OD600=0.5). BiFC signals from the AvrBsT and CaALDH1 interaction were visualized 40h after infiltration using a confocal laser scanning microscope (LSM 5 Exciter, Carl-Zeiss, Oberkochen, Germany) operated with LSM Imager. pSPYCE:*bZIP63* and pSPYNE:*bZIP63* were used as positive controls.

### Immunoblot analysis

Total soluble proteins were extracted from *N. benthamiana* leaves with 1ml denaturing buffer [50mM Tris-HCl (pH 8.8), 4M urea, 10mM sodium phosphate (pH 7.8), 250mM NaCl, 0.1% Nonidet P40, 1mM EDTA, and 0.5% SDS] per 0.5g leaf tissue. Insoluble debris was pelleted by centrifuging leaf extracts at 13 000 ×*g* for 20min at 4°C. For co-immunoprecipitation analyses, proteins were extracted in soluble buffer [50mM Tris-HCl (pH 8.8), 50mM NaCl, 10mM EDTA, 0.1% Triton X-100 and 2× protease inhibitor cocktail (Roche, Mannheim, Germany)]. Proteins in the supernatant were incubated with HA-agarose or cMyc-agarose (Sigma-Aldrich, St. Louis, USA) 4°C overnight. Proteins were resolved on 8% SDS-PAGE gels and transferred to PVDF membrane (GE Healthcare, Little Chalfont, United Kingdom). Proteins tagged with HA or cMyc epitopes were detected with anti-HA-peroxidase or anti-cMyc-peroxidase antibodies (Sigma), respectively.

### *Agrobacterium*-mediated transient expression

For subcellular localization analyses, *A. tumefaciens* strain GV3101 carrying pBIN35S:*GFP* or pBIN35S:*CaALDH1:GFP* was infiltrated into six-week-old *N. benthamiana* leaves. To confirm mitochondrial localization, leaf cells were counterstained with MitoTracker (Invitrogen). The lower epidermal cells were analysed 36↕48h after infiltration using a confocal laser scanning microscope (LSM 5 Exciter, Carl-Zeiss) operated with LSM Imager. For *Agrobacterium*-mediated transient expression, the *A. tumefaciens* strain GV3101 carrying pBIN35S:*avrBsT*, pBIN35S:*CaALDH1*, pBIN35S:*CaALDH1-E267A*, or pBIN35S:*CaALDH1-C301A* were infiltrated into six-week-old *N. benthamiana* leaves (Kim and Hwang, 2015).

### Virus-induced gene silencing

Tobacco rattle virus (TRV)-based virus-induced gene silencing (Liu *et al.*, 2002) was used to investigate *CaALDH1* loss-of-function in pepper plants. The non-conserved, C-terminal untranslated region of *CaALDH1* cDNA was PCR-amplified and cloned into the pCR2.1-TOPO vector. The gene-specific primers are listed in Supplementary Table S1. The cloned fragment was digested with *Eco*RI and inserted into pTRV2. The pTRV1, pTRV2:00, and pTRV2:*CaALDH1* constructs were transformed into *Agrobacterium* strain GV3101. An equal volume of pTRV1 *Agrobacterium* culture was mixed with one of the pTRV2 cultures before infiltration (OD600=0.2). The mixed cultures were infiltrated into cotyledons of pepper seedlings (Choi and Hwang, 2011). Four to five weeks after VIGS, *CaALDH1*-silenced plant leaves were used for quantitative RT-PCR and disease assays. The two other pepper *ALDH* genes (accession numbers *Capana09g000318* and *Capana09g000319*) that share high sequence identities of 95.19 and 86.14% with *CaALDH1*, respectively, were identified from a BLASTn search of *ALDHs* from the pepper genome sequence (http://peppersequence.genomics.cn) to use for the specific silencing test of *CaALDH1.*

### *Arabidopsis* transformation

Transgenic *Arabidopsis* plants expressing *CaALDH1* were generated by the floral-dip method (Clough and Bent, 1998). The *CaALDH1* coding region was inserted between the *cauliflower mosaic virus* (CaMV) 35S promoter and the *nos* terminator region in the pBIN35S binary vector. This construct was introduced into *Agrobacterium tumefaciens* GV3101 and used to transform *Arabidopsis thaliana* ecotype Columbia 0 (Col-0). Transformed seed stock was selected for kanamycin resistance by planting seeds on Murashige and Skoog (Duchefa, Haarlem, The Netherlands) agar plates containing 50mg l^-1^ kanamycin (Duchefa).

### Pathogen inoculation

*Xanthomonas campestris* pv. *vesicatoria* (*Xcv*) virulent Ds1 (EV, empty vector) and avirulent Ds1 (*avrBsT*) strains, and *Pseudomonas syringae* pv. *tomato* (*Pst*) DC3000 and DC3000 (*avrRpm1*) strains, were grown in YN (5g of yeast extract and 8g of nutrient broth l^-1^) or King’s B broth (10g of peptone, 1.5g of K2HPO4, 15g of glycerol, and 1mM MgSO4 l^-1^), respectively. The cultured bacteria were harvested and resuspended in 10mM MgCl2 solution. Bacterial growth in leaves was monitored at different time points after infiltration with *Xcv* (5×104 cfu ml^-1^) and *Pst* (105 cfu ml^-1^) using a needleless syringe.

*Hyaloperonospora arabidopsidis* (*Hpa*) Noco2 was grown on cotyledons of *Arabidopsis* seedlings at 16°C, 60% relative humidity, and a 14h photoperiod. *Hpa* conidia (5×104 conidia ml^-1^) were suspended in distilled tap water containing 0.05% Tween 20, and the inoculum suspension was sprayed onto cotyledons of seven-day-old *Arabidopsis* seedlings. The infected plants were incubated at 17°C in an environmentally controlled chamber.

### RNA gel-blot and real-time RT-PCR analyses

Total RNA was extracted from pepper plants using TRIzol reagent (Invitrogen, Carlsbad, CA, USA). RNA was resolved by agarose gel electrophoresis, transferred onto Hybond N+ membranes (GE Healthcare), and hybridized overnight with 32P-labelled *CaALDH1* cDNA. For real-time RT-PCR, 2 μg of RNA was used in a reverse-transcription reaction with MMLV reverse transcriptase (Enzynomics, Daejeon, Korea). Real-time RT-PCR was performed using iQ SYBR Green Supermix and iCycler iQ (Bio-Rad, Hercules, CA, USA). Gene-specific primer pairs used for real-time RT-PCR analysis are listed in Supplementary Table S1. The *C. annuum CaACTIN* transcript level was used for normalization of gene transcript levels. Relative expression levels were determined by comparing the calculated values with that of the uninoculated control.

### Aldehyde dehydrogenase activity assay

Aldehyde dehydrogenase (ALDH) activity was measured from crude plant extracts (Liu *et al.*, 2001). Leaf tissue was ground in liquid nitrogen and extracted with extraction buffer [0.1M HEPES buffer (pH 7.4), 1mM EDTA, 2mM DTT, and 0.1% Triton X-100]. The extract was centrifuged at 14 000 ×*g* for 20min, and the resulting supernatant was used as a crude enzyme extract. For each ALDH assay, 200mg of crude enzyme extract was added to a reaction mixture containing 1.5mM NAD (Sigma-Aldrich) and 0.1M sodium pyrophosphate buffer (pH 8.5) and the total volume was adjusted to 1.0ml with distilled water. The mixture was pre-incubated for 30 s before adding 17mM acetaldehyde to the mixture. The reaction was excited at 360nm, and NADH fluorescence emission was recorded at 460nm after 1min using a model Victor3 fluorescence spectrophotometer (Perkin Elmer, Massachusetts, USA). ALDH activity was expressed as nanomoles of NADH produced per minute per milligram of protein.

### Electrolyte leakage assay

Leaves of pepper or *N. benthamiana* plants were harvested at various time points after infiltration with *Xcv* or *Agrobacterium*, respectively. Leaf discs (0.5cm diameter) were excised with a cork borer and washed in 10ml of sterile double-distilled water for 30min with gentle agitation. Washed leaf discs were transferred to 20ml of sterile, double-distilled water and incubated for 2h at room temperature with gentle agitation. The ion conductivity of the leaf samples was measured using a Sension 7 conductivity meter (HACH, Loveland, CO, USA).

### Histochemistry

H_2_O_2_ accumulation was visualized by placing healthy or inoculated leaves in 1mg ml^-1^ 3,3’-diaminobenzidine (DAB) (Sigma-Aldrich) solution overnight (Thordal-Christensen *et al.*, 1997). Chlorophyll was cleared from the stained leaves by boiling in 95% ethanol. Cell death was monitored by trypan-blue staining of healthy or inoculated leaves (Koch and Slusarenko, 1990). Leaves were stained with lactophenol-trypan blue solution (10ml of lactic acid, 10ml of glycerol, 10g of phenol, and 10mg of trypan blue, dissolved in 10ml of distilled water), and destained in 2.5g ml^-1^ chloral hydrate solution. The samples were photographed using a digital camera (Olympus, Japan) mounted on a light microscope. Chlorosis, cell death, or phenolic compound accumulation in leaves was visualized using a hand-held UV lamp (UVP, CA, USA).

### H_2_O_2_ measurement

H_2_O_2_ accumulation in pepper and *Arabidopsis* leaves was quantified using xylenol orange (Gay *et al.*, 1999; Choi and Hwang, 2011). Xylenol orange assay reagent was freshly prepared by adding 500 μl of solution [25mM FeSO_4_ and 25mM (NH_4_)SO_4_ in 2.5M H_2_SO_4_] to 50ml of 125 μM xylenol orange in 100mM sorbitol. Eight leaf discs (0.5cm^2^) were floated on 1ml of distilled water in a microtube for 1h and centrifuged for 1min at 12 000 ×*g*, and 100 μl of supernatant was immediately added to 1ml of xylenol orange assay reagent. The mixture was incubated for 30min at room temperature. H_2_O_2_ was quantified by measuring the absorbance at 560nm using a DU 650 spectrophotometer (Beckman, Urbana, IL, USA) and compared with a standard curve for H_2_O_2_, which was generated by measuring a serial dilution of 100 nmol to 100 μmol of H_2_O_2_.

## Results

### CaALDH1 interacts with AvrBsT *in vitro* and *in planta*

**Fig. 1.**
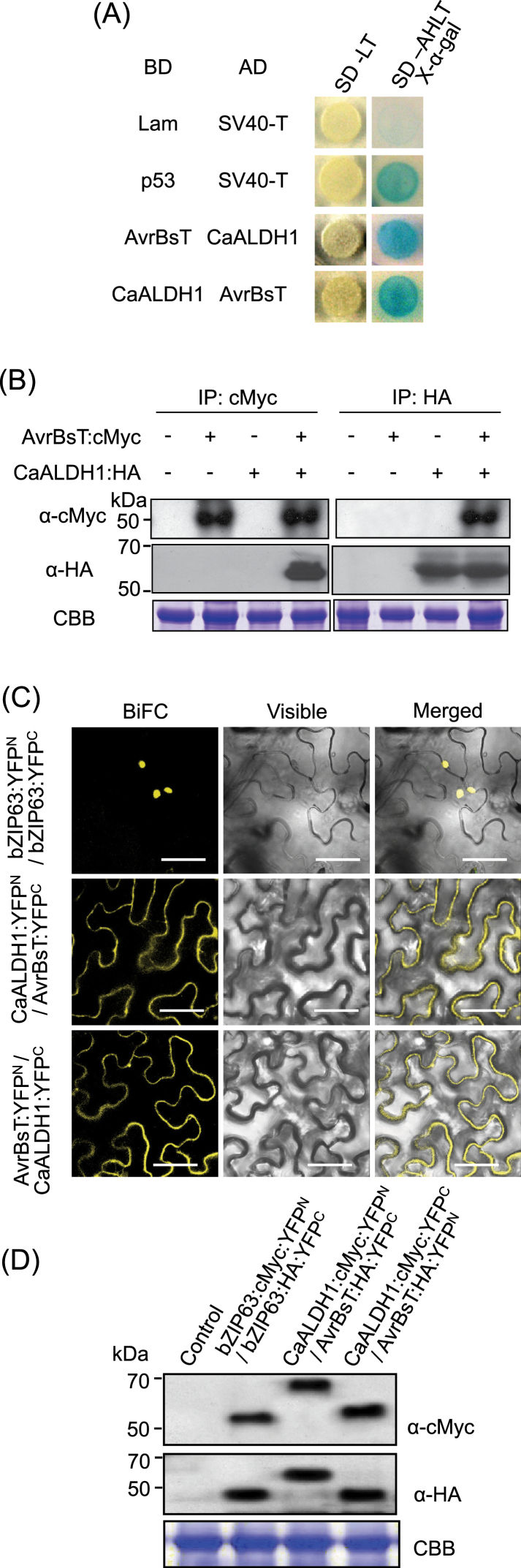
AvrBsT interacts with CaALDH1 in yeast and *in planta*. (A) Yeast two-hybrid assay. CaALDH1 and AvrBsT fused with GAL4 activation domain (AD) or binding domain (BD) were co-introduced into S*accharomyces cerevisiae* strain AH109, and reporter gene activation was monitored on synthetic dropout (SD)-AHLT (minus adenine, histidine, leucine, and tryptophan) medium containing X-α-gal. Combinations of Lam and p53 with SV40-T were used as negative and positive controls, respectively. (B) Co-immunoprecipitation analyses of transiently expressed CaALDH1:HA and AvrBsT:cMyc in *N. benthamiana* leaves. Extracted proteins were immunoprecipitated with α-cMyc or α-HA beads, and immunoblotted with α-cMyc and α-HA antibodies. (C) Bimolecular fluorescence complementation images of interactions between AvrBsT and CaALDH1 in *N. benthamiana* leaves. YFP fluorescence was visualized 30h after agroinfiltration using a confocal laser scanning microscope. bZIP63:YFP^N^ and bZIP63:YFP^C^ constructs were used as positive controls. Bars=50 µm. (D) Immunoblot analyses of YFP fusion proteins transiently expressed in *N. benthamiana* leaves. Protein loading was visualized by Coomassie brilliant blue (CBB) staining. (This figure is available in colour at *JXB* online.)

AvrBsT is an *Xcv* type III effector protein that triggers HR in pepper and *N. benthamiana* leaves (Kim *et al.*, 2010). A yeast two-hybrid screen was used to isolate molecular components that interact with AvrBsT (Fig. 1A). A prey library was generated by fusing the activation domain (AD) to pepper cDNAs synthesized from transcripts of pepper leaves undergoing hypersensitive response (HR), and AvrBsT was fused to the binding domain (BD) and used as bait to screen the library. One of the AvrBsT-interacting cDNAs encoded an aldehyde dehydrogenase protein (Supplementary Figs S1, S2). This clone was designated pepper aldehyde dehydrogenase 1 (*CaALDH1*) and was subjected to further characterization. *In silico CaALDH1* analyses determined that it encoded a C4-like member of aldehyde dehydrogenase family 2 (Supplementary Fig. S1). We compared the amino acid sequences of pepper CaALDH1 protein with those of other plant species. CaALDH1 shared 66–88% sequence identities with other ALDHs from *N. tomentosiformis* (88%, NtALDH; XP_009627665), *N. sylvestris* (88%, NsALDH; XP_009803645), grass (68%, LcALDH; ABO93608), *Arabidopsis* (67%, AtALDH; NP_566749), rice (66%, OsALDH; NP_001043453), and maize (66%, ZmALDH; AAL99608) (Supplementary Fig. S2A). However, any ALDH homologs of *N. benthamiana* were not searched from the NCBI’s GenBank database. Residues 267 and 301, glutamic acid and cysteine, respectively, are predicted to be essential for catalytic activity (Supplementary Figs S1, S2).

To investigate whether CaALDH1 interacts with AvrBsT in yeast, we swapped vectors and generated a DNA-binding domain (BD) fused with CaALDH1 and an activation domain (AD) fused with AvrBsT. We transformed these constructs into yeast with positive-control and negative-control vector pairs. AD-AvrBsT and BD- CaALDH1 interacted with each other and grew on selection media, as did BD-AvrBsT and AD-CaALDH1 (Fig. 1A). To investigate whether CaALDH1 and AvrBsT interact *in planta*, we performed co-immunoprecipitation (co-IP) analysis in *N. benthamiana* leaves using transiently expressed CaALDH1:HA and AvrBsT:cMyc (Fig. 1B). Extracted proteins were immunoprecipitated with α-cMyc or α-HA beads, separated by SDS-PAGE, and immunoblotted with α-cMyc and α-HA antibodies. Figure 1B shows that immunoblotting with α-Myc and α-HA antibodies detected AvrBsT:cMyc and CaALDH1:HA, respectively. Therefore, the co-IP analysis indicated that CaALDH1:HA physically interacted with AvrBsT:cMyc *in planta*.

Bimolecular fluorescence complementation (BiFC) assays were used to further investigate CaALDH1 and AvrBsT interaction *in planta* (Fig. 1C). CaALDH1 and AvrBsT were fused to the yellow fluorescent protein (YFP) N- and C-termini (Waadt *et al.*, 2008). *Agrobacterium* cells harbouring the corresponding constructs were mixed and co-infiltrated into *N. benthamiana* leaves. Confocal images of *N. benthamiana* epidermal cells detected YFP fluorescence in the cytoplasm but not in the nucleus (Fig. 1C), suggesting that CaALDH1 and AvrBsT interact with each other in the plant cytoplasm. As a positive control, the bZIP63:YFP transcription-factor construct exhibited fluorescence in the nucleus. Immunoblot assay results indicate that all YFP fusion proteins were transiently expressed with appropriate molecular weights in *N. benthamiana* leaves (Fig. 1D).

### *CaALDH1* expression is specifically induced in pepper leaves by avirulent *Xcv* infection

**Fig. 2.**
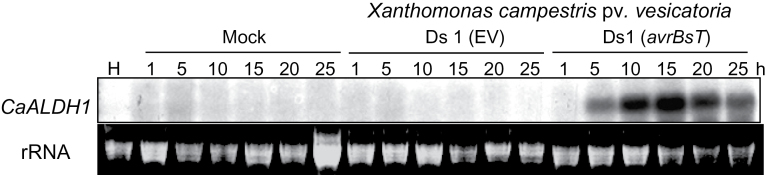
RNA gel blot analysis of *CaALDH1* expression in pepper leaves infected with virulent Ds1 (EV) or avirulent Ds1 (*avrBsT*) *Xcv*. RNA extracted from pepper plants was blotted to nylon membrane and hybridized with ^32^P-labelled *CaALDH1* probes. rRNA is shown as a loading control. H, healthy leaves; Mock, treated with 10mM MgCl_2_; EV, empty vector.

RNA gel blot analyses were used to investigate *CaALDH1* expression profiles during pepper plant interactions with *Xcv*. *CaALDH1* expression was strongly induced in pepper leaves during avirulent *Xcv* Ds1 (*avrBsT*) infection, compared with that of the mock or virulent *Xcv* Ds1 (EV) infection (Fig. 2). During incompatible interactions, *CaALDH1* expression distinctly increased 5h after inoculation, and the high expression level was maintained up to 25h after inoculation with avirulent *Xcv* Ds1 (*avrBsT*), indicating that *avrBsT* is required for induction of *CaALDH1* expression. In contrast, *CaALDH1* expression was not detected at any time point during mock or compatible interactions. These results indicate that *CaALDH1* expression is strongly and specifically induced during incompatible interactions with avirulent *Xcv* Ds1 (*avrBsT*).

### CaALDH1 is localized to the cytoplasm

**Fig. 3.**
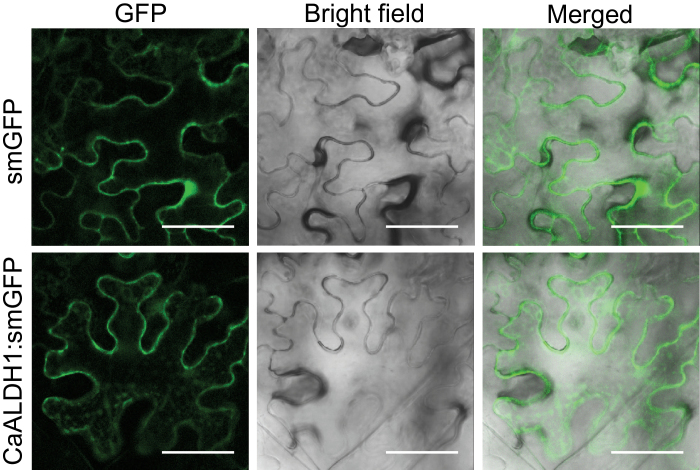
Subcellular localization of CaALDH1. *Agrobacterium*-mediated transient expression of *CaALDH1*:*smGFP* in *N. benthamiana* epidermal cells. GFP fluorescence was visualized using a confocal laser scanning microscope 48h after agroinfiltration. Bars=50 µm. (This figure is available in colour at *JXB* online.)

It has been proposed that human ALDHs be categorized as cytosolic ALDH1 and mitochondrial ALDH2 that are involved primarily in ethanol metabolism (Hsu *et al.*, 1988; Hsu *et al.*, 1989; Wang *et al.*, 1998; Vasiliou *et al.*, 1999). Family 1 ALDHs include the Class 1 ALDHs that are localized to the cytosol. Family 2 ALDHs are classified as the mitochondrial Class 2 ALDHs. To investigate CaALDH1 subcellular localization, a CaALDH1 fusion with soluble-modified green fluorescent protein (smGFP) (Davis and Vierstra, 1998) was transiently expressed in *N. benthamiana* leaves using agroinfiltration (Fig. 3). The control smGFP demonstrated that GFP fluorescence was ubiquitously distributed throughout the cell including the nucleus (Fig. 3). The CaALDH1:GFP fusion protein primarily localized to the cytoplasm (Fig. 3). The CaALDH1:GFP fusion proteins were also observed as small dots in the cytoplasm, but did not co-localize to the mitochondria (Supplementary Fig. S3). These results suggest that CaALDH1 belongs to the cytosolic Class1 ALDHs.

### Transient *CaALDH1* expression promotes *avrBsT*-triggered cell death, but not *Bax*-triggered cell death

**Fig. 4.**
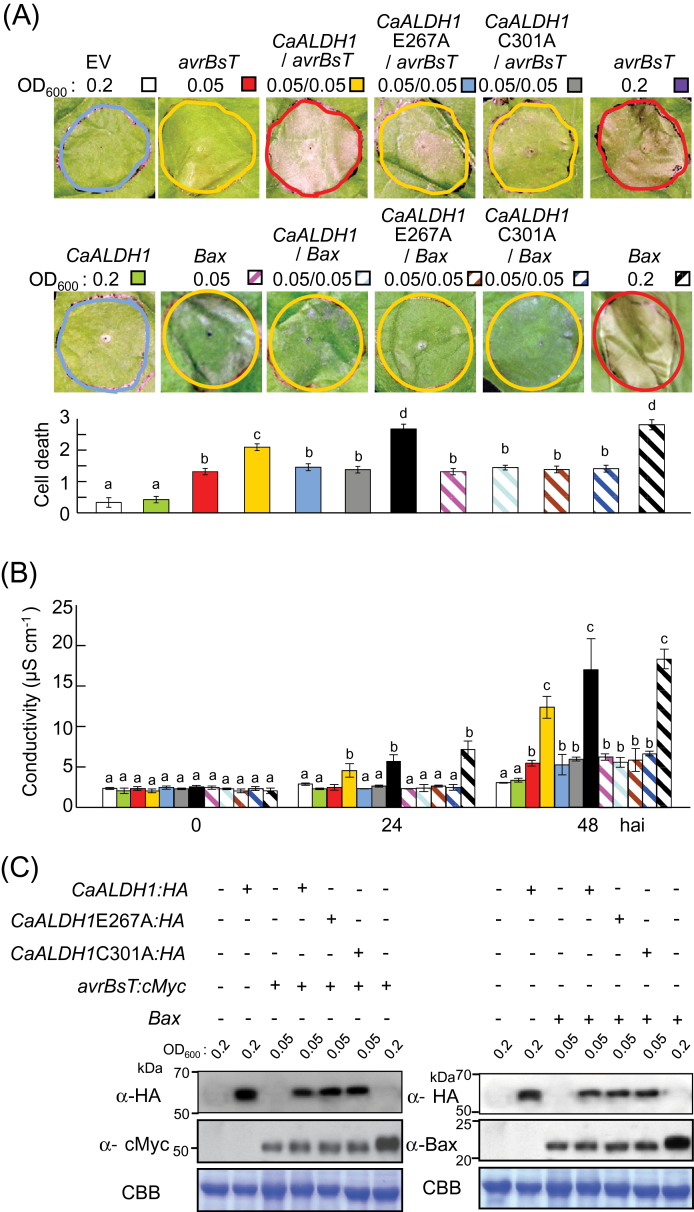
Transient *CaALDH1* expression promotes *avrBsT*-triggered cell death, but not *Bax*-triggered cell death. (A) Cell-death phenotypes and quantification in *N. benthamiana* leaves 2 days after infiltration with *Agrobacterium* carrying *CaALDH1, CaALDH1-E267A*, *CaALDH1-C301A, avrBsT* or *Bax* at different inoculum ratios. Cell death was quantified based on a 0−3 scale: 0, no cell death (<10%); 1, weak cell death (10–30%); 2, partial cell death (30–80%); and 3, full cell death (80–100%). Data are means ±SD from three independent experiments. Different letters indicate statistically significant differences (LSD, *P*<0.05). (B) Electrolyte leakage from leaf discs at different time points after infiltration with *Agrobacterium* carrying the indicated constructs at different inoculum ratios. Data are means ±SD from three independent experiments. Different letters indicate statistically significant differences (LSD, *P*<0.05). (C) Immunoblot analyses of transient expression of *CaALDH1*, *CaALDH1-E267A*, *CaALDH1-C301A*, *BAX* and *avrBs* 2 days after agroinfiltration. Protein loading was visualized by Coomassie brilliant blue (CBB) staining. (This figure is available in colour at *JXB* online.)

Co-infiltration with low titers (OD600=0.05) of *Agrobacterium* harbouring *CaALDH1* and *avrBsT* accelerated cell death in *N. benthamiana* leaves (Fig. 4). Figure 4A shows that transient *CaALDH1* expression (OD600=0.2) did not induce any cell death response, and transient *avrBsT* expression (OD600=0.05) did not induce typical HR cell death. However, coexpression of *CaALDH1* with *avrBsT* (OD600=0.05) induced a severe cell death response, similar to that induced by *avrBsT* agroinfiltration at OD600=0.2 (Fig. 4A). The Bcl-2-associated X protein (Bax) that induces hypersensitive cell death in *N. benathamiana* (Lacomme and Santa Cruz, 1999) was used as a positive control. As in *avrBsT* expression (OD600=0.2), *Bax* expression (OD600=0.2) induced typical HR cell death (Fig. 4A). By contrast, co-expression of *Bax* with *CaALDH1* resulting from infiltration with *Agrobacterium* at OD600=0.05 did not produce a cell death response. Co-expression of *avrBsT* or *Bax* with *CaALDH1-E267A* or *CaALDH1-C301A* (inactive mutations of *CaALDH1*) did not produce severe cell death in *N. benthamiana* leaves. These results indicate that CaALDH1 specifically interacts with AvrBsT to promote AvrBsT-triggered cell death.

The extent of cell death was quantified by measuring electrolyte leakage from *N. benthamiana* leaf discs transiently expressing *CaALDH1* and/or *avrBsT* or *Bax* (Fig. 4B). Control leaves expressing empty vector and leaves transiently expressing *CaALDH1* did not exhibit electrolyte leakage. These results were consistent with the observed cell death phenotypes. However, leaf tissues co-expressing *CaALDH1* with *avrBsT,* but not with *Bax,* at the low titers (OD600=0.05) exhibited high levels of electrolyte leakage, similar to that of leaves transiently expressing high levels of *avrBsT* or *Bax* at 24 and 48h after agroinfiltration (OD600=0.2). Leaves co-expressing inactive *CaALDH1* mutants with *avrBsT* or *Bax* did not show high levels of electrolyte leakage. Immunoblot analyses confirmed that CaALDH1, CaALDH1-E267A, CaALDH1-C301A, AvrBsT and Bax proteins were transiently expressed in *N. benthamiana* leaves (Fig. 4C). Collectively, these results indicate that *CaALDH1* expression promotes *avrBsT*-triggered hypersensitive cell death response.

### CaALDH1 exhibits aldehyde dehydrogenase activity *in planta*

**Fig. 5.**
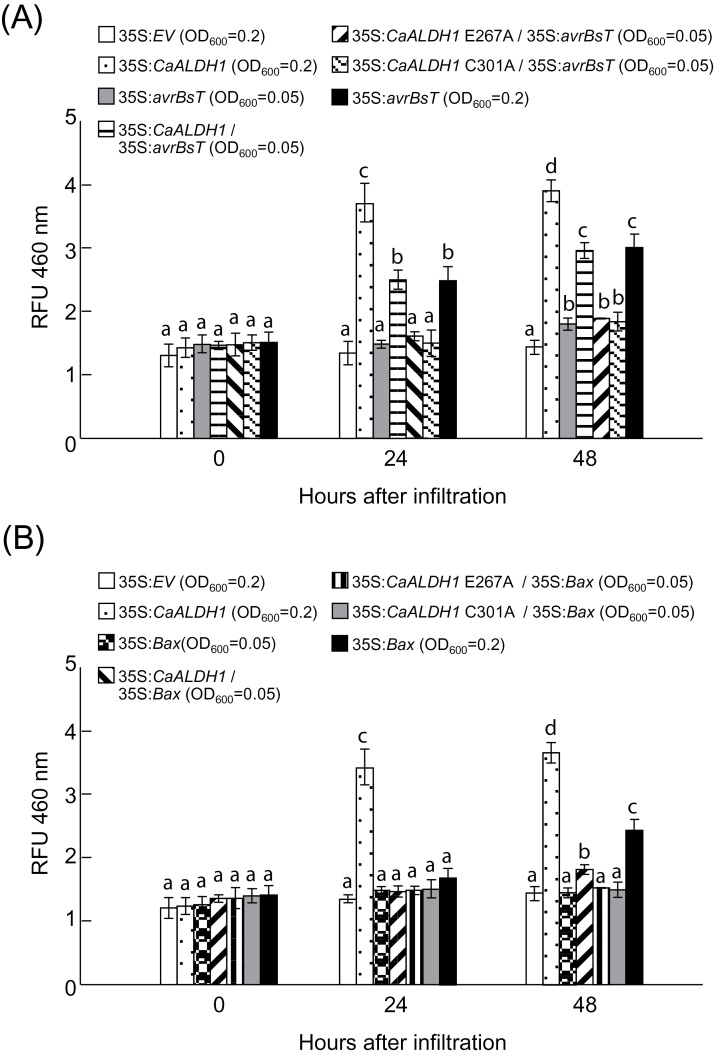
Aldehyde dehydrogenase activity assay. (A) Coexpression of *CaALDH1*, *CaALDH1-E267A*, or *CaALDH1-C301A* with *avrBsT*. (B) Coexpression of *CaALDH1, CaALDH1-E267A*, or *CaALDH1-C301A* with *Bax*. *N. benthamiana* leaves were infiltrated with *Agrobacterium* carrying the indicated constructs at different inoculum ratios. ALDH activity in crude extracts was assayed at 0, 24 and 48h after agroinfiltration. RFU 460nm, relative fluorescence units at 460nm.

Crude extracts prepared from *N. benthamiana* leaves agroinfiltrated with empty vector control, *CaALDH1*, *CaALDH1-E267A*, *CaALDH1-C301A*, and *avrBsT* were assayed for aldehyde dehydrogenase activity using acetaldehyde as substrate (Fig. 5A). Transient *CaALDH1* expression (OD600=0.2) caused a 3-fold increase in ALDH activity compared to that of the empty vector control 24h after agroinfiltration. Transient *avrBsT* expression (OD600=0.05) caused a slight elevation in ALDH activity 48h after agroinfiltration. However, *avrBsT* and *CaALDH1* co-expression (OD600=0.05) caused a 2-fold increase in ALDH activity 24h after agroinfiltration, similar to that of *avrBsT* expression (OD600=0.2). Co-expression of inactive *CaALDH1* mutants (E267A or C301A) with *avrBsT* (OD600=0.05) did not affect ALDH activity. Crude extracts prepared from *N. benthamiana* leaves infiltrated with empty vector control, *CaALDH1*, inactive *CaALDH1* mutants (E267A or C301A), and *Bax* were assayed for ALDH activity using acetaldehyde as the substrate (Fig. 5B). Co-expression of *CaALDH1* or inactive *CaALDH1* mutants (E267A or C301A) with *Bax* (OD600=0.05) did not affect ALDH activity. These results indicate that *CaALDH1* expression induces ALDH activity, which is required for *avrBsT*-triggered plant cell death.

### *CaALDH1* silencing reduces *avrBsT*-mediated resistance, ROS burst, and cell death during *Xcv* infection

**Fig. 6.**
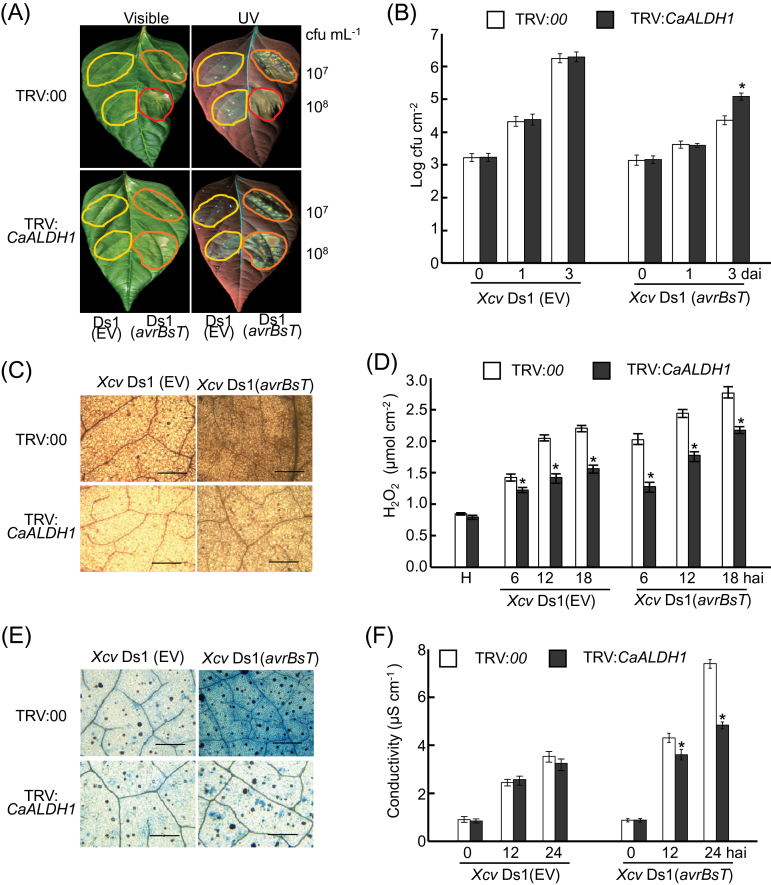
Enhanced susceptibility of *CaALDH1*-silenced pepper leaves to avirulent Ds1 (*avrBsT*) or virulent Ds1 (EV) *Xcv* infection. (A) Enhanced disease symptoms on *CaALDH1*-silenced pepper leaves. Yellow line, symptoms visible only under UV illumination; orange line, visible symptoms; red line, complete cell death. (B) Bacterial growth in leaves at 0, 1 and 3 days after *Xcv* infiltration. *Xcv* Ds1 (EV) and Ds1 (*avrBsT*) (5×10^5^ cfu ml^-1^) were infiltrated into empty-vector control (TRV:00) and *CaALDH1*-silenced (TRV:*CaALDH1*) leaves. (C) DAB staining and (D) quantification of H_2_O_2_ at different time points after infiltration with *Xcv* Ds1 (*EV*) and Ds1 (*avrBsT*) (5×10^7^ cfu ml^-1^). (E) Trypan blue staining and (F) electrolyte leakage from leaves infiltrated with *Xcv* Ds1 (EV) and Ds1 (*avrBsT*) (5×10^7^ cfu ml^-1^). Trypan blue staining was performed 24h after infiltration. Asterisks indicate statistically significant differences (*t*-test; *P*<0.05). Data are mean values ±SD from three independent experiments with four replicates each. EV, empty vector. (This figure is available in colour at *JXB* online.)

The tobacco rattle virus (TRV)-based virus-induced gene silencing (VIGS) technique was used to generate *CaALDH1* loss-of-function pepper plants (Liu *et al.*, 2002). To reduce the nonspecific silencing effect, the non-conserved, C-terminal untranslated region (UTR) of *CaALDH1* cDNA was cloned into the TRV2 vector to specifically silence *CaALDH1*. RT-PCR analysis showed that *CaALDH1* was effectively silenced in pepper plants infected with *Xcv* (Supplementary Fig. S4). Leaves of non-silenced (TRV*:00*) and *CaALDH1*-silenced (TRV:*CaALDH1*) pepper were inoculated with virulent *Xcv* Ds1 (EV) or avirulent Ds1 (*avrBsT*) strains (107 and 108 cfu ml^-1^). Avirulent *Xcv* infection (108 cfu ml^-1^) of non-silenced (TRV*:00*) leaves caused HR and complete necrosis two days after inoculation; however, *CaALDH1* silencing greatly reduced HR to avirulent *Xcv* infection (Fig. 6A). Reduced cell death in *CaALDH1*-silenced leaves was evident under UV illumination (Fig. 6A). Avirulent *Xcv* Ds1 (*avrBsT*) growth in *CaALDH1*-silenced leaves was approximately 7-fold higher than that in non-silenced (TRV*:00*) leaves three days after inoculation (Fig. 6B).

Early defence responses such as ROS (H_2_O_2_) accumulation and cell death response were analysed in non-silenced (TRV*:00*) and *CaALDH1*-silenced pepper leaves during *Xcv* infection (Fig. 6C, D). H_2_O_2_ production and cell death were visualized by DAB (Fig. 6C) and trypan-blue staining (Fig. 6E), respectively. Significantly reduced H_2_O_2_ accumulation and cell death responses were observed in *CaALDH1*-silenced leaves inoculated with *Xcv.* Xylenol orange assay and ion conductivity measurements were used to quantify H2O2 production and cell death, respectively. *CaALDH1* silencing significantly reduced H2O2 production in pepper leaves during virulent *Xcv* Ds1 and avirulent *Xcv* Ds1 (*avrBsT*) infection (Fig. 6D). Cell death triggered by avirulent *Xcv* Ds1 (*avrBsT*) infection was significantly reduced, with a corresponding reduction in electrolyte leakage from infected pepper leaf tissue (Fig. 6F). Collectively, these results indicate that *CaALDH1* is required for *avrBsT*-mediated resistance to *Xcv* infection.

### *CaALDH1* silencing attenuates ALDH activity and defence-responsive gene expression

**Fig. 7.**
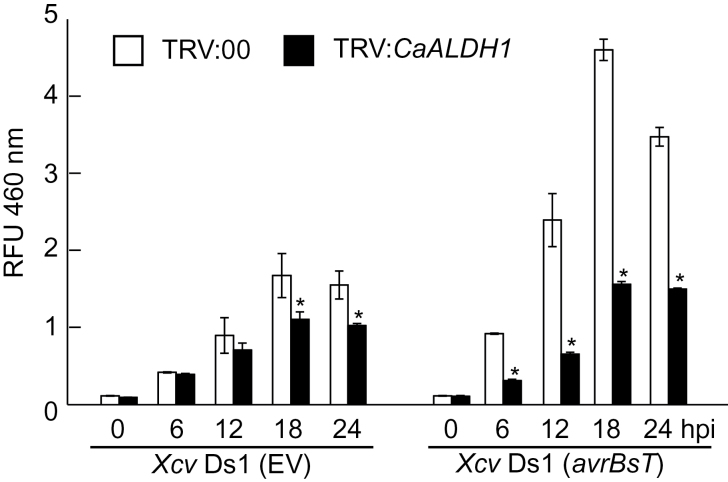
Decreased aldehyde dehydrogenase activity in *CaALDH1*-silenced pepper leaves infiltrated with *Xcv* Ds1 (*EV*) and Ds1 (*avrBsT*) (5×10^5^ cfu ml^-1^). ALDH activity in pepper leaf crude extracts was analysed at different time points after *Xcv* infiltration. Asterisks indicate statistically significant differences (*t*-test; *P*<0.05). Data are mean values ±SD from three independent experiments. RFU 460nm, relative fluorescence units at 460nm; EV, empty vector.

ALDH activity was analysed in leaves of non-silenced (TRV*:00*) and silenced (TRV:*CaALDH1*) pepper plants during *Xcv* infection. Crude extracts prepared from pepper leaves 0, 6, 12, 18 and 24h after inoculation with *Xcv* were analysed for ALDH activity using acetaldehyde as the substrate (Fig. 7). Avirulent *Xcv* Ds1 (*avrBsT*) infection induced substantially higher ALDH activity in non-silenced (TRV*:00*) leaves than that of virulent *Xcv* Ds1 (EV) infection. *CaALDH1* silencing significantly reduced induction of ALDH activity during virulent *Xcv* Ds1 (EV) infection. Avirulent *Xcv* Ds1 (*avrBsT*) infection induced 2↕4-fold higher ALDH activity in empty-vector control leaves than in *CaALDH1*-silenced leaves 6↕24h after inoculation.

**Fig. 8.**
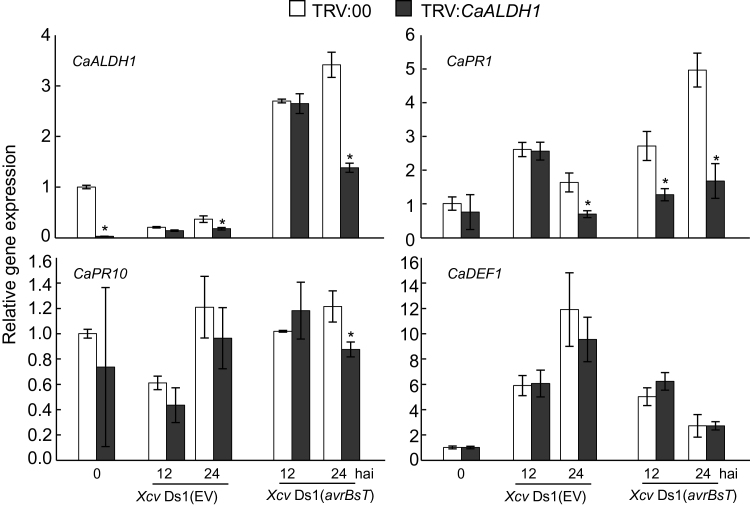
Quantitative real-time RT-PCR analysis of defence-related gene expression in empty-vector control (TRV:*00*) and *CaALDH1*-silenced (TRV:*CaALDH1*) pepper leaves infiltrated with *Xcv* Ds1 (EV) and Ds1 (*avrBsT*) (5×10^7^ cfu ml^-1^). *CaPR1*, PR1; *CaPR10*, PR10; *CaDEF1*, defensin1. Expression levels of *Capsicum annuum CaACTIN* were used to normalize defence-related gene expression levels. Asterisks indicate statistically significant differences (*t*-test; *P*<0.05). Data are mean values ±SD from three independent experiments. EV, empty vector.

Real-time RT-PCR analyses were performed using gene-specific primer pairs for *CaALDH1*, *CaPR1* (*PR1*) (Kim and Hwang, 2000), *CaDEF1* (*defensin*) (Do *et al.*, 2004), and *CaPR10* (*PR10*) (Choi *et al.*, 2012) during *Xcv* infection (Fig. 8) to determine the effects of *CaALDH1* silencing on the expression of defence-related genes at early infection stages in pepper. *CaALDH1* silencing in pepper leaves significantly attenuated expression of salicylic acid (SA)-dependent defence genes *CaPR1* and *CaPR10*, but not the jasmonate-related gene *CaDEF1*, during avirulent *Xcv* infection. These results indicate that *CaALDH1* is involved in SA-dependent defence signalling during compatible and incompatible interactions of *Xcv* with pepper.

### Enhanced defence response of *CaALDH1*-overexpressing *Arabidopsis* to *P. syringae* pv. *tomato* and *H. arabidopsidis*

**Fig. 9.**
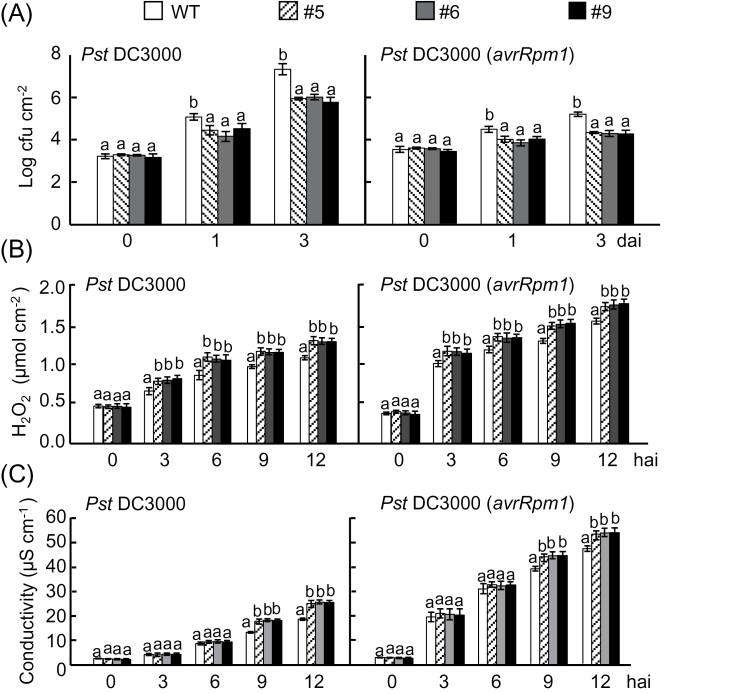
Enhanced resistance of *CaALDH1*-overexpressing *Arabidopsis* plants to *Pst* DC3000 and DC3000 (*avrRpm1*). (A) Bacterial growth in leaves of wild-type and *CaALDH1*-overexpressing plants at 0, 1 and 3 days after inoculation (5×10^5^ cfu ml^-1^). (B) H_2_O_2_ quantification at different time points after inoculation (5×10^7^ cfu ml^-1^)_._ (C) Electrolyte leakage measurement. Wild-type and *CaALDH1*-overexpressing leaves were infiltrated with *Pst* DC3000 and DC3000 (*avrRpm1*) (5×10^7^ cfu ml^-1^), and electrolyte leakage was monitored at the indicated time points. Data are mean values ±SD from three independent experiments with four replicates each. Different letters indicate significant differences at different time points (Fisher’s least significant differences; *P*<0.05).

The effect of ectopic *CaALDH1* expression on disease resistance was analysed by generating transgenic *CaALDH1*-overexpressing (OX) *Arabidopsis* plants using the floral-dip method (Clough and Bent, 1998). Three independent *CaALDH1*-OX transgenic lines (#5, #6, and #9) were confirmed by RT-PCR to constitutively express *CaALDH1* (Supplementary Fig. S5). Four-week-old wild-type and *CaALDH1*-OX *Arabidopsis* plants were inoculated with *Pst* DC3000 and DC3000 (*avrRpm1*) (5×105 cfu ml^-1^) (Fig. 9A). Viable bacterial counts in leaves increased approximately 10-fold more in wild-type plants than in *CaALDH1*-OX plants three days after inoculation of *Pst* DC3000. The *Pst* DC3000 (*avrRpm1*) bacterial titers in *CaALDH1*-OX plants were significantly lower than those in wild-type plants. These data indicate that *CaALDH1* overexpression enhances *Arabidopsis* defence responses and attenuates *Pst* DC3000 and DC3000 (*avrRpm1*) growth *in planta*.

*CaALDH1* overexpression strengthened early defence responses such as ROS burst and cell death response in *Arabidopsis* leaves during *Pst* infection. *CaALDH1*-OX lines exhibited higher H_2_O_2_ accumulation levels in response to *Pst* infection compared with that of wild-type (Fig. 9B). H_2_O_2_ bursts during 3–12h after inoculation with *Pst* were significantly higher in *CaALDH1*-OX *Arabidopsis* leaves compared with those in wild-type leaves. The cell death response was quantified as the level of electrolyte leakage from discs of infected leaves (Fig. 9C). *CaALDH1* overexpression led to significantly higher electrolyte leakage from leaf tissues 9–12h after *Pst* infection compared with that in wild-type leaves (Fig. 9C). In general, infection with avirulent *Pst* DC3000 (*avrRpm1*) induced higher ROS burst and electrolyte leakage from *Arabidopsis* leaves compared with that of virulent *Pst* DC3000 infection.

**Fig. 10.**
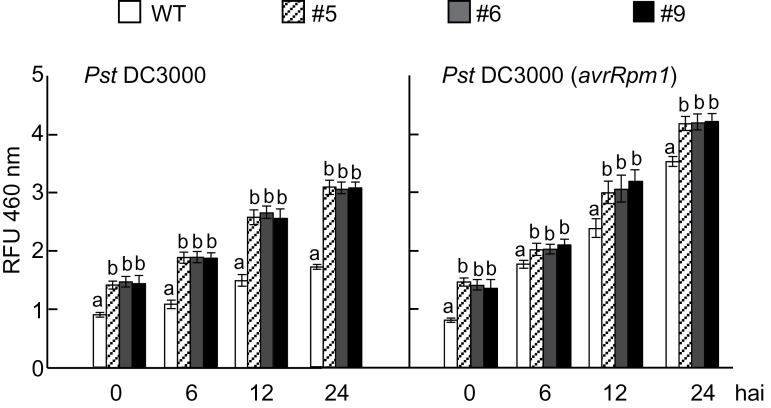
Enhanced aldehyde dehydrogenase activity in leaves of *CaALDH1*-overexpressing *Arabidopsis* lines in response to *Pst* DC3000 and DC3000 (*avrRpm1*) (5×10^7^ cfu ml^-1^) infection. ALDH activity in pepper leaf crude extracts was analysed at the indicated time points after infiltration with *Pst*. RFU 460nm, relative fluorescence units at 460nm.

ALDH activity levels were analysed in leaves of wild-type and *CaALDH1*-OX *Arabidopsis* plants inoculated with *Pst* DC3000 (5×107 cfu ml^-1^) (Fig. 10). When challenged with *Pst* DC3000 and DC3000 (*avrRpm1*), *CaALDH1*-OX plants exhibited significantly higher ALDH activity compared with that of wild-type leaves during infection. *Pst* DC3000 (*avrRpm1*) infection induced higher ALDH activity compared with that of *Pst* DC3000 infection in *Arabidopsis* leaves.

**Fig. 11.**
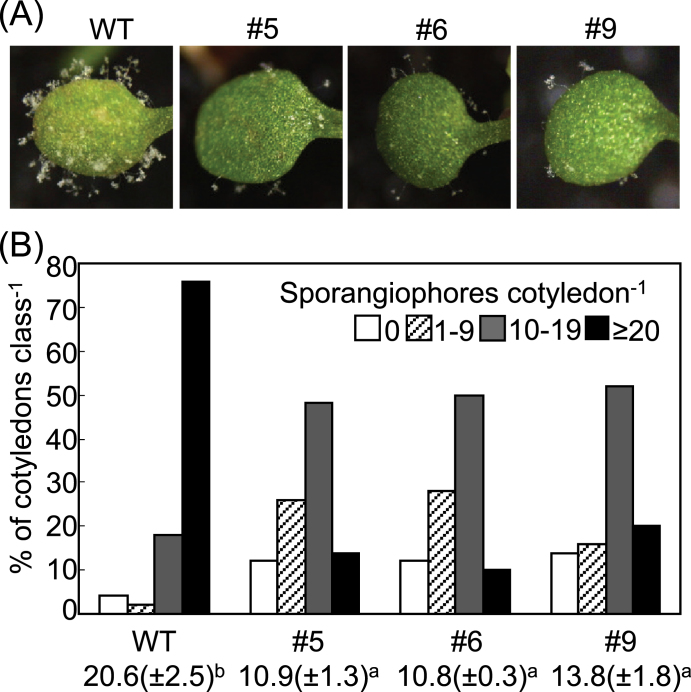
Enhanced resistance of *CaALDH1*-overexpressing *Arabidopsis* plants to *Hpa* Noco2 infection. (A) Disease symptoms on cotyledons of wild-type and *CaALDH1*-overexpressing plants 7 days after inoculation with *Hpa* Noco2. (B) Number of sporangiophores per cotyledon of wild-type and *CaALDH1*-overexpressing plants at 5 days after inoculation. Data were quantified based on the number of sporangiophores counted per cotyledon as follows: 0, no sporulation; 1−9, light sporulation; 10−19, medium sporulation; ≥20, heavy sporulation. More than 50 cotyledons of wild-type and *CaALDH1*-overexpressing plant lines were counted. Average numbers of sporangiophores are given below the chart. Data represent mean values ±SD from three independent experiments. Different letters indicate statistically significant differences (LSD, *P*<0.05). (This figure is available in colour at *JXB* online.)

Seven-day-old wild-type and transgenic *Arabidopsis* seedlings were inoculated with a conidiospore suspension of *Hpa* isolate Noco2 (Fig. 11). *CaALDH1*-OX lines exhibited reduced susceptibility to *Hpa* infection (Fig. 11A). A significant decrease in the number of sporangiophores was observed in cotyledons of *CaALDH1*-OX seedlings compared with the observed sporangiophore proliferation in wild-type plants five days after inoculation (Fig. 11B).

## Discussion

Pepper leaves infected with the virulent *Xcv* Ds1 develop susceptible lesions, which appear water-soaked and turn yellow three days after inoculation (Lee and Hwang, 1996). *Xcv* type III effector AvrBsT induces hypersensitive cell death and defence responses in pepper, but not in tomato (Kim *et al.*, 2010). Introduction of AvrBsT into *Xcv* Ds1 rendered the strain avirulent to pepper plants (Kim *et al.*, 2010). *Xcv* Ds1 (*avrBsT*) infection induced hypersensitive response (HR) in pepper leaves. AvrBsT translocates into pepper cells via the *Xcv* type III secretion system, and triggers a HR during infection (Escolar *et al.*, 2001). AvrBsT also triggers hypersensitive cell death in *N. benthamiana* (Orth *et al.*, 2000; Kim *et al.*, 2010). AvrBsT activates effector-triggered immunity (ETI) in *Arabidopsis thaliana* Pi-0 plants (Cunnac *et al.*, 2007). We recently identified that pepper CaSGT1 (suppressor of the G2 allele of *skp1*) interacted with AvrBsT (Kim *et al.*, 2014) and promoted AvrBsT-triggered hypersensitive cell death in a phosphorylation-dependent manner. Our previous findings support the possibility that AvrBsT may interact with some host receptors related to plant cell death. Here, we demonstrated that pepper aldehyde dehydrogenase 1 (CaALDH1) interacted with AvrBsT *in planta*, and promoted AvrBsT-triggered cell death and defence responses in plants. The compelling data prompted an investigation into the role of CaALDH1 in AvrBsT-triggered cell death and defence response.

NAD(P)+-dependent aldehyde dehydrogenase is involved in the detoxification of stress-generated aldehydes (Kirch *et al.*, 2004). Aldehyde dehydrogenases are generally localized to mitochondria or cytosol (Sophos and Vasiliou, 2003; Kirch *et al.*, 2004). The *Arabidopsis* ALDH2C4 shares 98% identity with CaALDH1, and is predicted to accumulate in the cytosol (Skibbe *et al.*, 2002). We show that CaALDH1:smGFP accumulates primarily in the cytosol. ALDHs scavenge toxic aldehydes generated from abiotic stresses. *ALDH3I1* and *ALDH7B4* overexpression enhances tolerance to drought, salinity, and oxidative stress in *Arabidopsis* (Sunkar *et al.*, 2003; Kotchoni *et al.*, 2006). Our transient co-expression analyses indicate that CaALDH1 promotes AvrBsT-triggered cell death, but not Bax-triggered cell death, in *N. benthamiana*. The Bax protein has been known as a general cell death trigger in *N. benthamiana* (Lacomme and Santa Cruz, 1999). Bax is reported to promote apoptosis through its action on mitochondria and downstream activation of caspases in mammals (Arnoult *et al.*, 2003). Yeast two-hybrid, bimolecular fluorescence complementation and co-immunoprecipitation assays revealed the physical interaction between CaALDH1 and AvrBsT in yeast and *in planta*. These results support the notion that CaALDH1 may be directly involved in promoting AvrBsT-triggered cell death. Heterologous *CaALDH1* coexpression with *avrBsT* in *N. benthamiana* significantly enhances *avrBsT*-triggered cell death. Cell death induction is dependent on aldehyde dehydrogenase activity of CaALDH1. However, co-expression of inactive *CaALDH1* mutants (E267A and C301A) did not promote *avrBsT*-triggered cell death. Collectively, these results suggest that the CaALDH1-complex formation contributes positively to the promotion of AvrBsT-triggered cell death.

RNA gel-blot analysis indicates that *CaALDH1* is strongly induced in pepper leaves by avirulent *Xcv* (*avrBsT*) infection. ALDHs are predicted to play an important role for defence responses by detoxifying stress-generated aldehydes (Kirch *et al.*, 2004). Loss-of-function analyses of *CaALDH1* via virus-induced gene silencing shows that CaALDH1 aldehyde dehydrogenase activity is important for *R* gene-mediated defence responses in pepper. *CaALDH1*-silenced pepper plants exhibit significantly reduced ALDH activity in leaves. *CaALDH1* silencing attenuated ALDH activity and ROS burst, and significantly reduced cell death and defence responses to avirulent *Xcv* (*avrBsT*) infection. *CaALDH1*-silenced plants also exhibit significantly reduced expression of defence-related genes such as *CaPR1* (Kim and Hwang, 2000) and *CaPR10* (Choi *et al.*, 2012), which have been identified in pepper as a defence-response marker and a cell-death regulator, respectively.

*CaALDH1* overexpression in *Arabidopsis* suppressed *Pst* DC3000 and DC3000 (*avrRpm1*) growth. *CaALDH1*-OX plants accumulate significantly higher ROS levels during *Pst* infection, ultimately resulting in enhanced cell death. The ROS burst mediates cell-defence responses in pepper plants (Choi *et al.*, 2007). These results support a potential functional role of CaALDH1 in plant immunity to *Pst* infection. This role is observed during obligate biotrophic downy mildew infection. *CaALDH1* overexpression strongly suppresses *Hpa* Noco2 growth. These results support the proposal that CaALDH1 activity effectively enhances defence response to biotrophic oomycete infection. Together, these data suggest that CaALDH1 is required to trigger basal defence and *R* gene-mediated resistance to microbial pathogens in plants.

We integrate these data to propose a working model for the role of the CaALDH1 and AvrBsT complex in plant cell death and defence signalling in response to microbial pathogens (Supplementary Fig. S6). *Xcv* Ds1 (*avrBsT*) secretes the type III effector AvrBsT into host plant cells to modulate immune signalling during infection. Expression of *avrBsT* in *Xcv* Ds1 rendered the strain avirulent to pepper plants (Kim *et al.*, 2010). Infection of pepper leaves with *Xcv* Ds1 (*avrBsT*) expressing *avrBsT* triggers hypersensitive response (HR) accompanied by strong H_2_O_2_ generation, callose deposition and defence-marker gene expressions. Rapid induction of pepper *CaALDH1* is triggered by *Xcv* (*avrBsT*) challenge. *CaALDH1* expression induces ALDH activity, ROS burst, cell death response as well as expression of salicylic acid (SA)-dependent defence genes in plants. CaALDH1 interacts with AvrBsT in the plant cell cytoplasm to promote AvrBsT-triggered cell death and defence responses. The CaALDH1 and AvrBsT complex promotes ALDH activity, which may promote ROS burst and induce expression of some PR genes such as *CaPR1* (Kim and Hwang, 2000) and *CaPR10* (Choi *et al.*, 2012). The cumulative effect of CaALDH1 activity enhances HR-like cell death and defence responses. CaALDH1 together with AvrBsT may function upstream of ROS-mediated cell-death signalling during *Xcv* (*avrBsT*) infection. Collectively, the results presented in this study suggest that CaALDH1 acts as a positive regulator that promotes AvrBsT-triggered cell death and defence responses in plants.

## Supplementary material

Supplementary data are available at *JXB* online.

Supplementary Fig. S1. Nucleotide and deduced amino acid sequences of pepper *CaALDH1* cDNA.

Supplementary Fig. S2. Comparison of aldehyde dehydrogenase homologues in plants.

Supplementary Fig. S3. CaALDH1 does not localize to the mitochondria.

Supplementary Fig. S4. RT-PCR analysis of expression of *CaALDH1, Capana09g000318* and *Capana09g000319* in leaves of empty-vector control (TRV:*00*) and *CaALDH1*-silenced (TRV:*CaALDH1*) pepper plants.

Supplementary Fig. S5. RT-PCR analysis of *CaALDH1* expression in leaves of four-week-old wild-type and *CaALDH1*-overexpressing *Arabidopsis* lines.

Supplementary Fig. S6. Proposed model for the functional role of CaALDH1-AvrBsT complex in plant cell death and defence signalling.

Supplementary Table S1. Gene-specific primers used in this study.

Supplementary Data

## Abbreviations:

ABA: abscisic acid
ALDH: aldehyde dehydrogenase
Bax: Bcl-2-associated X protein
BiFC: bimolecular fluorescence complementation
Co-IP: co-immunoprecipitation
DAB: 3,3’-diaminobenzidine
ETI: effector-triggered immunity
HR: hypersensitive response
Hpa: *Hyaloperonospora arabidopsidis*
LB: Luria-Bertani
NLR: nucleotide-binding leucine-rich repeat
OX: over-expression
PAMP: pathogen-associated molecular pattern
PCD: programmed cell death
PCR: polymerase chain reaction
PR: pathogenesis-related
PRRs: pattern-recognition receptors
Pst: *Pseudomonas syringae* pv. *tomato*
R: resistance
ROS: reactive oxygen species
SA: salicylic acid
SGT1: suppressor of the G2 allele of *skp1*
smGFP: soluble-modified green fluorescent protein
TRV: tobacco rattle virus
VIGS: virus-induced gene silencing
Xcv: *Xanthomonas campestris* pv. *vesicatoria*.
