# Supplementary material for: Pepper aldehyde dehydrogenase CaALDH1 interacts with Xanthomonas effector AvrBsT and promotes effector-triggered cell death and defence responses
Source: J Exp Bot. 2015 Apr 6;66(11):3367–80. doi: 10.1093/jxb/erv147 (PMC4449550; doi:10.1093/jxb/erv147)
Supplement: Supplementary Data [file supp_66_11_3367__index.html]

Pepper aldehyde dehydrogenase CaALDH1 interacts with Xanthomonas effector AvrBsT and promotes effector-triggered cell death and defence responses — Pepper aldehyde dehydrogenase CaALDH1 interacts with Xanthomonas effector AvrBsT and promotes effector-triggered cell death and defence responses — Supplementary Data 

# Pepper aldehyde dehydrogenase CaALDH1 interacts with *Xanthomonas* effector AvrBsT and promotes effector-triggered cell death and defence responses

## Supplementary Data

Data files

**Files in this Data Supplement:**

- Supplementary Data - Supplementary Data
