# Supplementary material for: Pepper aldehyde dehydrogenase CaALDH1 interacts with Xanthomonas effector AvrBsT and promotes effector-triggered cell death and defence responses
Source: J Exp Bot. 2015 Apr 6;66(11):3367–80. doi: 10.1093/jxb/erv147 (PMC4449550; doi:10.1093/jxb/erv147)
Supplement: Supplementary Data [file supp_erv147_jexbot144519_file001.pdf]

Pepper aldehyde dehydrogenase CaALDH1 interacts with *Xanthomonas* effector AvrBsT and promotes effector-triggered cell death and defense responses

Nak Hyun Kim and Byung Kook Hwang

```
acagtcaaatttaatttgccatttcccctgtgtgaagcttttaaaagaaaaaaaaaaaaa   60
ATGGTGGAATCAAATGGAAATTTAGAAAATCATGTCCAAATTCCAAAAATTAAGTTCACC  120
 M  V  E  S  N  G  N  L  E  N  H  V  Q  I  P  K  I  K  F  T
AAGCTCTTTATCAATGGAGAATTCGTCGATTCCGTTTCAGGAAATACGTTCGAGACGATA  180
 K  L  F  I  N  G  E  F  V  D  S  V  S  G  N  T  F  E  T  I
GATCCAAGAAATGGGGAGATAATTGCAAGAATTAGTGAAGGTGACAAAGAGGATATTAAT  240
 D  P  R  N  G  E  I  I  A  R  I  S  E  G  D  K  E  D  I  N
TTGGCTGTCAAAGCTGCTCGTGAAGCTTTCGATAATGGCCCTTGGCCTCGTTTAGCACCT  300
 L  A  V  K  A  A  R  E  A  F  D  N  G  P  W  P  R  L  A  P
TCGGAGAGGAGAAGGATAATGCTGAAGTTCGCGGACTTAATCGTAGAAAATGCAGAGGAA  360
 S  E  R  R  R  I  M  L  K  F  A  D  L  I  V  E  N  A  E  E
ATTGCAGCATTGGATGCAATGGATGCAGGAAAGTTATTTGCTCCTTTAAAGAACATGGAA  420
 I  A  A  L  D  A  M  D  A  G  K  L  F  A  P  L  K  N  M  E
ATCCCGTCAGCAGCAGAAACTATTCGTTATTATGCTGGTGCAGCCGATAAAATTCATGGG  480
 I  P  S  A  A  E  T  I  R  Y  Y  A  G  A  A  D  K  I  H  G
ACGACTCTTAAAATGTCACGCGAGATACAAGGGTACACTTTGCTCGAGCCAATTGGTGTT  540
 T  T  L  K  M  S  R  E  I  Q  G  Y  T  L  L  E  P  I  G  V
GTCGGACACATTATTCCTTGGAATTTCCCGACACAGATGTTTACTCTGAAGGTTGGCCCT  600
 V  G  H  I  I  P  W  N  F  P  T  Q  M  F  T  L  K  V  G  P
GCGCTAGCAGCCGGTTGTACTATGATTGTCAAACCTGCTGAACAAACTCCTCTTTCCGCT  660
 A  L  A  A  G  C  T  M  I  V  K  P  A  E  Q  T  P  L  S  A
CTCTACTACGCTCACTTGGCTAAGCAGGCAGGTGTTCCCGATGGAGTGATCAATGTCGTA  720
 L  Y  Y  A  H  L  A  K  Q  A  G  V  P  D  G  V  I  N  V  V
ACAGGATTTGGATCTACTGCTGGTGCTGCACTTTGCTCGCATATGCACGTAGACAAGATA  780
 T  G  F  G  S  T  A  G  A  A  L  C  S  H  M  H  V  D  K  I
AGCTTCACAGGCTCGACAGAAGTTGGACGACTAGTAATGCAGGCTGCAGCGTTAAGCAAT  840
 S  F  T  G  S  T  E  V  G  R  L  V  M  Q  A  A  A  L  S  N
TTAAAACCTGTCTCGCTAGAGCTGGGAGGGAAGTCGCCTTTTATAGTATTCGATGATGTA  900
 L  K  P  V  S [L  E  L  G  G  K  S  P] F  I  V  F  D  D  V
GATGTTGACAAAGTTGCACCACTTGCTCTTATCGGAATCCTATACAACAAGGGAGAAATT  960
 D  V  D  K  V  A  P  L  A  L  I  G  I  L  Y  N  K  G  E  I
TGTGTGGCTGGATCTCGTCTTTTCATCCAAGAAGGAATTTACGATAAATTTGTCAAGAAA 1020
 C  V  A  G  S  R  L  F  I  Q  E  G  I  Y  D  K  F  V  K  K
TTGGAGGCGATGGCAAAAACATGGGTTGTTGGCGATCCTTTTGATCCAAATTCTCATCAA 1080
 L  E  A  M  A  K  T  W  V  V  G  D  P  F  D  P  N  S  H  Q
GGACCTCAAGTTGATAAAAAACAGTTCGAGAGAGTACTTTCATACATTGAACATGGCAAG 1140
 G  P  Q  V  D  K  K  Q  F  E  R  V  L  S  Y  I  E  H  G  K
AAGGAGGGAGCGAAATTGCTAACCGGAGGCAAGGCATTGGATAGGAAAGGTTATTTCATC 1200
 K  E  G  A  K  L  L  T  G  G  K  A  L  D  R  K  G  Y  F  I
GAGCCAACCATATTCATTGATGTTGAAGACGATATGAAAATTGCAAAAGAAGAAATATTT 1260
 E  P  T  I  F  I  D  V  E  D  D  M  K  I  A  K  E  E  I  F
GGACCTGTTTTATCAGTAATGAAGTTCAAGACGGTAGAGGAGGTAATCAAGAGAGCTAAC 1320
 G  P  V  L  S  V  M  K  F  K  T  V  E  E  V  I  K  R  A  N
TGTACAAATTATGGGACTAGCAGCGGGTGTAATGACCAATGCCTTGAACATTGCTACACA 1380
 C  T  N  Y  G  T  S  S  G  C  N  D  Q  C  L  E  H  C  Y  T
GTTTCAAGATCGATACGTGCTGGTGTCATCTGGATAAACTGTTACTTTGCTTTCGATGCG 1440
 V  S  R  S  I  R  A  G  V  I  W  I  N  C  Y  F  A  F  D  A
GATTGTCCGTATGGAGGATACAAAAGCAGTGGATTTGAAAGAGATTTGGGAATGGAAGGA 1500
 D  C  P  Y  G  G  Y  K  S  S  G  F  E  R  D  L  G  M  E  G
CTTCATAAGTATTTTCAAGTTAAATCTGTAGCTACTCCTATTTACAACTCTCCTTGGCTG 1560
 L  H  K  Y  F  Q  V  K  S  V  A  T  P  I  Y  N  S  P  W  L
TAAatttgcagaaaacaaagtgccaacatattagatttttattactattgccatagtatt 1620
 *
ttcatgttataaaaaaaaaaaaaaaaaaaaaaaaaaaaaaaaaaaaaaaa           1670
```

Supplementary Fig. S1. Kim and Hwang

(A)

```
CaALDH1 MVESNGNLENHVQIP--KIKFTKLFINGEFVDSVSGNTFETIDPRNGEIIARISEGDKED 58
NtALDH  MVQSNGNLECHFQIP--KIKFTQLFINGEFVDSVSGNTFETIDPRNEEVIARIAEGEKAD 58
NsALDH  MVKSNGNLESHFQIP--KIKFTQLFINGEFVDSVSGNTFETIDPRNEEVIARIAEGDKAD 58
LcALDH  MAAAN--SDKGFEVPELDIKFTKLFINGQFVDAASGKTFETRDPRTGEVIAMIAEGDKAD 58
AtALDH  MENGKCNGATTVKLP--EIKFTKLFINGQFIDAASGKTFETIDPRNGEVIATIAEGDKED 58
OsALDH  MAAANGGDSKGFEVPKLEIKFTKLFINGRFVDAVSGKTFETRDPRTGEVIAKIAEGDKAD 60
ZmALDH  MATANGSSKGPFEVPKVEVRFTKLFIDGKFVDAVSGKTFETRDPRTGEVIASIAEGGKAD 60

CaALDH1 INLAVKAAREAFDNGPWPRLAPSERRRIMLKFADLIVENAEEIAALDAMDAGKLFAPLKN 118
NtALDH  IDLAVKAAREAFDNGPWPRLSPLERRNIMLKFADLIIENAEEIAALDSMDAGKLFAAGKN 118
NsALDH  IDLAVKAAREAFDNGPWPRLSPMERRNIMLKFADLIIENAEEIAALDAMDAGKLFAAGKT 118
LcALDH  IDLAVKAAREAFDNGPWPRMSGCARARIMHKFADLVDQHVEKLAALDTVDAGKLFQMGKL 118
AtALDH  VDLAVNAARYAFDHGPWPRMTGFERAKLINKFADLIEENIEELAKLDAVDGGKLFQLGKY 118
OsALDH  IDLAVKAAREAFDHGPWPRMSGFARGRILHKFADLVEQHVEELAALDTVDAGKLFAMGKL 120
ZmALDH  VDLAVKAAREAFDNGPWPRMTGYERGRILHRFADLIDEHVEELAALDTVDAGKLFAVGKA 120

CaALDH1 MEIPSAAETIRYYAGAADKIHGTTLKMSRE-IQGYTLLEPIGVVGHIIPWNFPTQMFTLK 177
NtALDH  TDIPSAAQFMRYYAGAADKIHGTTLKMSRD-IQGYTLLEPIGVVGHIIPWNFPTQMFVMK 177
NsALDH  IDVPSAAQFMRYYAGAADKIHGTTLKMSRD-IQGYTLLEPIGVVGHIIPWNFPTQMFVMK 177
LcALDH  MDIPGGANLLRYYAGAADKIHGETLKMARP-LHGYTLKEPVGVVGHIVPWNYPTTMFFFK 177
AtALDH  ADIPATAGHFRYNAGAADKIHGETLKMTRQSLFGYTLKEPIGVVGNIIPWNFPSIMFATK 178
OsALDH  VDIPGGANLLRYYAGAADKVHGETLKMARP-CHGYTLKEPVGVVGHIVPWNYPTTMFFFK 179
ZmALDH  RDIPGAAHLLRYYAGAADKVHGATLKMAQR-MHGYTLKEPVGVVGHIVPWNYPTTMFFFK 179

CaALDH1 VGPALAAGCTMIVKPAEQTPLSALYYAHLAKQAGVPDGVINVVTGFGSTAGAALCSHMHV 237
NtALDH  VGPALAAGCTMVVKPAEQTPLSALYYAQLAKQAGVPDGVINVVTGFGSTAGAALCSHMDV 237
NsALDH  VGPALAAGCTMVVKPAEQTPLSALYYAQLAKQAGVPDGVINVVTGFGSTAGAALCSHMDV 237
LcALDH  VSPALAAGCTMVVKPAEQTPLSALFYAHLAKEAGIPDGVLNVVPGFGPTAGAAIASHMDI 237
AtALDH  VAPAMAAGCTMVVKPAEQTSLSALFYAHLSKEAGIPDGVLNIVTGFGSTAGAAIASHMDV 238
OsALDH  ASPALAAGCTMVVKPAEQTPLSALFYAHLAKLAGVPDGVLNVVPGFGPTAGAAISSHMDI 239
ZmALDH  VGPALAAGCAVVVKPAEQTPLSALFYAHLAREAGVPAGVLNVVPGFGPTAGAAVAAHMDV 239

                                        ========              ----
CaALDH1 DKISFTGSTEVGRLVMQAAALSNLKPVSLELGGKSPFIVFDDVDVDKVAPLALIGILYNK 297
NtALDH  DKISFTGSTEVGRLVMQAAALSNLKPVSLELGGKSPFIVFDDVDVEKVAPLALIGILYNK 297
NsALDH  DKISFTGSTEVGRLVMQAAALSNLKPVSLELGGKSPFIVFDDVDVDKVAPLAMIGILYNK 297
LcALDH  DKISFTGSTEVGRLVMQAAAMSNLKPVSLELGGKSPIIVFDDADVDMAVNLVNMATYTNK 297
AtALDH  DKVSFTGSTDVGRKIMQAAAASNLKKVSLELGGKSPLLIFNDADIDKAADLALLGCFYNK 298
OsALDH  DKVSFTGSTEVGRLVMEAAAKSNLKPVSLELGGKSPVIVFDDADLDTAVNLVHMASYTNK 299
ZmALDH  DKVSFTGSTEVGRLVMRAAAESNLKPVSLELGGKSPVIVFDDADLDMAVNLVNFATYTNK 299

        --------
CaALDH1 GEICVAGSRLFIQEGIYDKFVKKLEAMAKTWVVGDPFDPNSHQGPQVDKKQFERVLSYIE 357
NtALDH  GEVCVAGSRLFIQEGIYDKFLKKLEEITKTWVVGDPFDTNSHQGPQVDKKQFERVLSYIE 357
NsALDH  GEVCVAGSRLFIQEGIYDKFVKKLVEITKTWVVGDTFDPNSHQGPQVDKKQFERVLSYIE 357
LcALDH  GEICVAGTRIYVQEGIYDAFVKKSVELAKKSVVGDPFNPNVHQGPQVDKDQYEKVLKYIN 357
AtALDH  GEICVASSRVFVQEGIYDKVVEKLVEKAKDWTVGDPFDSTARQGPQVDKRQFEKILSYIE 358
OsALDH  GEICVAGSRIYVQEGIYDAFVKKATEMAKKSVVGDPFNPRVHQGPQIDKEQYEKILKYID 359
ZmALDH  GEICVAGTRIYVQEGIYDEFVKKAAELASKSVVGDPFNPSVSQGPQVDKDQYEKVLRYID 359

CaALDH1 HGKKEGAKLLTGGKALDRKGYFIEPTIFIDVEDDMKIAKEEIFGPVLSVMKFKTVEEVIK 417
NtALDH  HGKREGATLLTGGNALDRKGYFIEPTIFTGVEDHMIIAKEEIFGPVLSVMKFKTVEEVIK 417
NsALDH  HGKREGATLLTGGNALDRKGYFVEPTIFTDVEDHMTIAKEEIFGPVLSVMKFKTVEEVIK 417
LcALDH  VGKSEGATLLTGGKPCGDKGYYIEPTIFTDVKDDMSIAQEEIFGPVMALMKFKTVEEVIR 417
AtALDH  HGKNEGATLLTGGKAIGDKGYFIQPTIFADVTEDMKIYQDEIFGPVMSLMKFKTVEEGIK 418
OsALDH  IGKREGATLVTGGKPCGENGYYIEPTIFTDVKEEMSIAQEEIFGPVMALMKFKTVEEAIQ 419
ZmALDH  IGKREGATLVTGGKPCGDKGYYIEPTIFTDVKDDMTIAQDEIFGPVMALMKFKTVEEVIQ 419

CaALDH1 RANCTNYGTSSGCNDQCLEHCYTVSRSIRAGVIWINCYFAFDADCPYGGYKSSGFERDLG 477
NtALDH  RANCTQYGLAAGVMTNNLNIANTVSRSIRAGVIWINCYFAFDPDCPYGGYKSSGFERDLG 477
NsALDH  RANCTQYGLAAGVMTNNLNIANTVSRSIRAGVIWINCYFAFDPDCPYGGYKSSGFERDLG 477
LcALDH  KANNTRYGLAAGVVTKNIDTMNTVSRSVRAGVIWVNCYFAFDPDAPFGGCKMSGFGKDMG 477
AtALDH  CANNTKYGLAAGILSQDIDLINTVSRSIKAGIIWVNCYFGFDLDCPYGGYKMSGNCRESG 478
OsALDH  KANSTRYGLAAGIVTKNIDVANTVSRSIRAGAIWINCYLGFDPDVPFGGYKMSGFGKDMG 479
ZmALDH  KANNTRYGLAAGIVTKNIDVANTVSRSIRAGAIWINCYFAFDPDAPFGGYKMSGFGKDMG 479

CaALDH1 MEGLHKYFQVKSVATPIYNSPWL- 500
NtALDH  MEGLHKYLQVKSVATPIYNSPWL- 500 (88%)
NsALDH  MEGLHKYLQVKSVATPIYNSPWL- 500 (88%)
LcALDH  TDALDKYLHTKTVVTPLYNTPWL- 500 (68%)
AtALDH  MDALDNYLQTKSVVMPLHNSPWM- 501 (67%)
OsALDH  MDALEKYLHTKAVVTPLYNTPWL- 502 (66%)
ZmALDH  MDALDKYLQTKTVVTPLYNTPWL- 502 (66%)
```

(B)

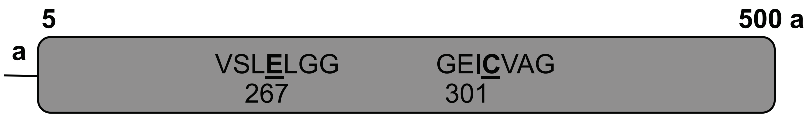

Supplementary Fig. S2. Kim and Hwang

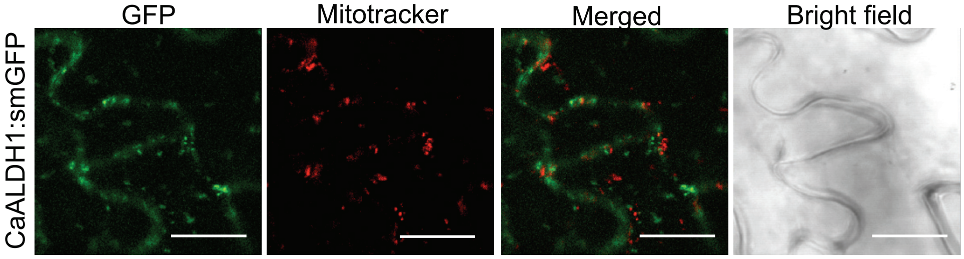

Supplementary Fig. S3. Kim and Hwang

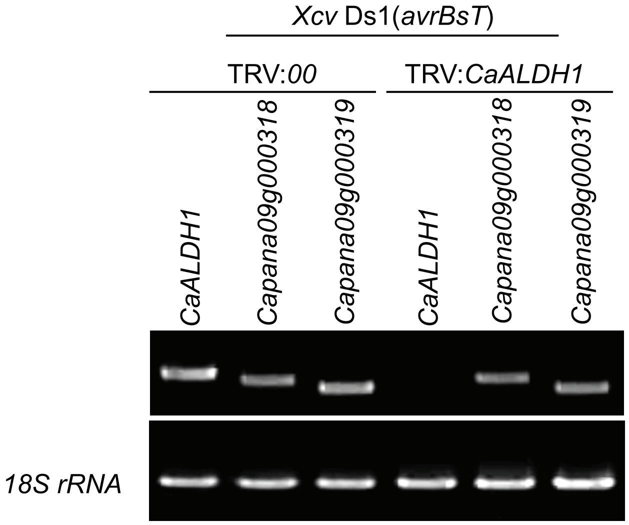

Supplementary Fig. S4. Kim and Hwang

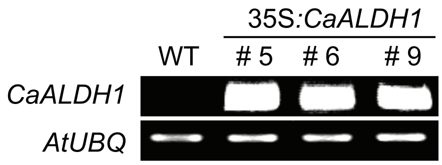

Supplementary Fig. S5. Kim and Hwang

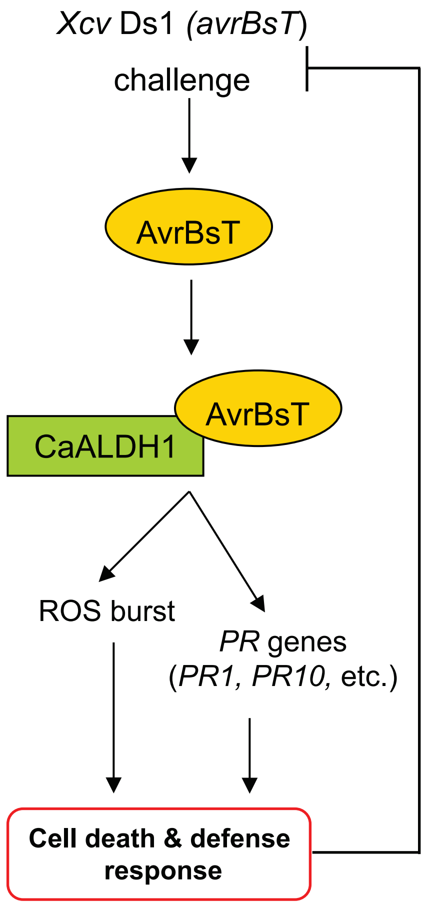

Supplementary Fig. S6. Kim and Hwang

Supplementary Table S1. Gene-specific primers used in this study		
Analysis	Gene	Forward and reverse primer sequence
Yeast two-hybrid	*CaALDH1* F	5'-GAGAGAGACCATGGAGATGGTGGAATCAAATGGAAAT-3'
	CaALDH1 R	5'-GAGAGAGAGTCGACCAGCCAAGGAGAGTTGTAAATAG-3'
	*avrBs*T F	5'-GAGACATATGATGAAGAATTTTATGCGTTCACTTG-3'
	avrBsT R	5'-GAGACTCGAGTGATTCAATAGTTTTCCTAATTTT-3'
VIGS	*CaALDH1* vigs F	5'-TAAATTTGCAGAAAACAAAG-3'
	CaALDH1 vigs R	5'-TTTATAACATGAAAATACTA-3'
BiFC	*avrBsT* F	5'-GAGAACTAGTATGAAGAATTTTATGCGTTCACTTG-3'
	avrBsT R	5'-GAGACTCGAGTGATTCAATAGTTTTCCTAATTTTCC-3'
	CaALDH1 F	5'-GAGAGAGATCTAGAATGGTGGAATCAAATGGAAAT-3'
	CaALDH1 R	5'-GAGAGAGAGTCGACCAGCCAAGGAGAGTTGTAAATAG-3'
pBIN35S and pBIN35S:GFP	*CaALDH1* F	5'-GAGAGAGATCTAGAATGGTGGAATCAAATGGAAAT-3'
	CaALDH1 R	5'-GAGAGAGAGTCGACCAGCCAAGGAGAGTTGTAAATAG-3'
	Bax F	5'-GAGAGATCTAGAATGGACGGGTCCGGGGAGCA-3'
	Bax R	5'-GAGAGACTCGAGTCAGCCCATCTTCTTCCAGA-3'
Quantitative real-time RT-PCR	*CaALDH1* 500 F	5'-ATGGCGGGAAAGGGTGAAGGTC-3'
	CaALDH1 500 R	5'-GAATAATGTGTCCGACAACACCAA-3'
	Ca*PR1* F	5'-CAGGATGCAACACTCTGGTGG-3'
	Ca*PR1* R	5'-ATCAAAGGCCGGTTGGTC-3'
	Ca*PR10* F	5'-TGACCTTTGTCGAAGGTGGT-3'
	Ca*PR10* R	5'-GTAAGTAAACTTGTTATATTC-3'
	Ca*DEF1* F	5'-CAAGGGAGTATGTGCTAGTGAGAC-3'
	Ca*DEF1* R	5'-TGCACAGCACTATCATTGCATAC-3'
	CaACTIN F	5'-AAGCTCTCCTTTGTTGCTGTT-3'
	CaACTIN R	5'-GACTTCTGGGCATCTGAATCT-3'
RT-PCR	*CaALDH1* F	5'-ATGGTGGAATCAAATGGAAAT-3'
	CaALDH1 R	5'-CAGCCAAGGAGAGTTGTAAATAG-3'
	Capana09g00031	5'-ATGCATAACAAAAATTTTATTGGGTC-3'
	Capana09g00031	5'-TTACAGCCAAGGAGAGTTGTAAATAG-3'
	Capana09g00031	5'-ATGGTGCAATCTAAAGATAATTCAGA-3'
	Capana09g00031	5'-TTACAGCCAAGGAGAGTTGTAAATAG-3'
